# Supplementary material for: The health care and life sciences community profile for dataset descriptions
Source: PeerJ. 2016 Aug 16;4:e2331. doi: 10.7717/peerj.2331 (PMC4991880; doi:10.7717/peerj.2331)
Supplement: Supplemental Information 1 [file peerj-04-2331-s001.docx]

9 Use Cases

9.1 Marshfield Use Case

Marshfield Clinic is one of the largest physician group practices in the United States, who employs approximately 780 physicians representing 84 different specialities and 6,500 additional staff working on the main campus in Marshfield or one of 52 regional clinics serving the population of Wisconsin and the upper peninsula of Michigan. Integral to clinical practice, Marshfield Clinic Research Foundation (MCRF) conducts clinical and biomedical research projects.

Marshfield is a member of the Health Maintenance Organization Research Network (HMORN). To enable population-based health and health care research across 16 members in HMORN, Marshfield participates in a standardized federated data system called Virtual Data Warehouse (VDW). VDW enables the query to be distributed to different sites. The query result from each site is summarized at each site and then combined to be returned to the requester. In such a scenario, a description of the original query, the database that was queried, and a description of the query results would be useful for keeping track of the provenance of the query results. Useful metadata include human readable titles, detailed descriptions, a list of individuals responsible, date and times for when the database was compiled, the query was issued, the results obtained, as well as licensing for the database and results, keywords describing the database, the format of the query and results, and the version of the database.

Marshfield also participates in a clinical pharmacogenomics consortium called eMERGE-PGx. A goal of eMERGE-PGx is to discover novel associations between genotypes and pharmacogenomics responses. The more data accumulated from different participating sites, the higher the statistical power to detect novel associations. Standardized metadata description, especially descriptors of license conditions and rights, will ensure transparencies in genetic research and data sharing. With proper metadata available for each data set, pharmacogenomics discovery can be made by agent-based algorithms and proper reuse of data.

9.2 Metaome Transcriptomics Use Case

Gene expression data analysis has emerged as a powerful approach for understanding biology. RNA-seq, microarrays and qPCRs are some of the common approaches for analyzing gene expression. To understand gene function, it is often necessary to analyze gene expression over various experimental conditions. It is important that, from a large corpus of transcriptomics datasets, users are able to retrieve datasets that match specific experimental criteria. Further, based on the experiment metadata, the datasets can be enriched with relevant information such as related mutations, cell lines and phenotypes.

A well-structured and standardized dataset description is imperative for this operation. Such an approach of slicing large corpus of transcriptomics data, based on biological parameters and experimental conditions is recognized by our customers as a strong base for gene expression analytics. A structured approach to describing datasets combined with gene expression analytics could lead to applications such as better classification of tumor samples and drug-repurposing.

9.3 Radiotherapy Research Use Case

MAASTRO Clinic is a radiotherapy clinic with approximately 200 employees and 60 researchers. Several use case scenarios at MAASTRO make use of distributed heterogeneous data, where data discovery plays a critical role.

The first use case scenario involves a MAASTRO researcher wants to perform a retrospective study using previously collected routine clinical care data. Often, the necessary data is spread across several databases and data sources, requiring knowledge of several data schemas and interfaces in order to retrieve data. Ideally, a Research Data Archive (RDA) would present a single access point for researchers to find many types of data for their research including: patient demographic data, treatment and planning data such as CT images, tumor volume, dose, and fractionation (i.e. doses divided across 'fractions'). The RDA is being built from a combination of a data warehouse and SPARQL federation and will contain a catalogue of all data sources, some of which will be data that has been analyzed in specific publications. In cases where data has been calculated by a program, the data should include provenance such as the program and version which was used to generate it. A self-maintaining catalogue (i.e. dynamically generated from dataset descriptions) would help to keep the RDA manageable as it grows to a larger scale, as well as lower the cost of adding data sources and maintenance of tools for data access.

In projects such as [euroCAT](http://www.eurocat.info/) and [EURECA](http://eurecaproject.eu/), MAASTRO needs to facilitate data sharing with external partners. In EuroCAT, a system for machine learning has been built up based on a principle of a uniform data interface that enables machine learning algorithms to 'visit' data at the hospital and clinic. This circumvents the security and legal problems that arise when sending clinical data outside the walls of the hospital. In this case, each hospital could use dataset descriptions to make data discovery possible for other partners. The same principle can be applied to biobanks.

In the EURECA project, a legal and technical framework has been created that enables clinical care and clinical research data to be shared via a Center for Data Protection (CDP). However, as the data collection grows, it becomes steadily more difficult to find data based on attributes of interest. Therefore, a standardized dataset description will enable project partners to make better use of the data by enabling data discovery.

MAASTRO shares data from [CancerData.org](https://www.cancerdata.org/) and would like to make data discoverable via SPARQL.

9.4 Computational Network Biology

**9.4.1 Biological Network Data Users**

The Cytoscape software for network visualization and analysis [[Cytoscape](https://www.w3.org/TR/hcls-dataset/#Cytoscape)] and the GeneMANIA software for gene function prediction (http://genemania.org/) depend on loading biological network and related data from numerous and diverse sources to support various types of analysis. Cytoscape is a stand alone workbench application and data loading is driven by users and GeneMANIA primarily accesses data using an automated build process. Both systems will benefit from knowledge of the following aspects of a data set:

- license - generally needed to support commercial users who need to separate commercial from open data
- data source short name (for GUI display purposes), full name (may go in a tooltip) and description
- homepage URL, PMIDs (find out more information about the data set/data source)
- example data URL - useful to help browse contents of an online data source, e.g. an example pathway database record
- production date - needed to communicate to users about how current the data is
- download date - needed to communicate to users about how current the data is
- version - needed to communicate to users about how current the data is
- update cycle - frequency of update, useful for build systems to figure out how often to check for data updates
- dataset statistics. Useful to gauge the overall size of the data. Ideally this would be the number of genes/protein/molecules and the number of interactions, broken down into type of interaction would be even better

For GeneMANIA, to fully automate discovery of new data sets (a true intelligent agent), we would need to know the data type (e.g. protein-protein interaction, text mined co-citations, pathways, gene expression), the organism, the types of gene identifiers and the number of genes covered by a given number of interactions.

For Cytoscape, most users would also be interested to know how others have used the data, as otherwise, they would not know what to do with the list of data sets (at least if there was no further categorization).

**9.4.2 Pathway Commons and Pathguide.org**

The goal of Pathway Commons (http://www.pathwaycommons.org/about/) is to collect all publicly available biological pathway information and make it easily and widely available. Pathguide (http://www.pathguide.org/) tracks over 540 databases containing pathway related information. This tracking website is currently manually updated, but it would be great if it could be automatically updated by downloading metadata files from each database. In addition to the above metadata useful for GeneMANIA and Cytoscape, the nature of the data source in terms of originality is important for Pathway Commons, as originally curated information is desired, not redundant copies of data from meta-databases. Pathguide terms this “primary” (originally curated or predicted) or “secondary” (collected from other sources). An example Pathguide record for GeneMANIA is at<http://pathguide.org/fullrecord.php?organisms=all&availability=all&standards=all&order=alphabetic&DBID=334>.

9.5 Safety Information Evaluation and Visual Exploration ("SIEVE")

AstraZeneca ("AZ") Patient Safety Science wanted to improve retrieval of clinical trial data and biometric assessments across studies. Traditionally, evaluation of clinical trials data across studies required manual intervention to deliver desired datasets. A proposal titled Safety Information Evaluation and Visual Exploration ("SIEVE") was sponsored by Patient Safety Science. This took the form of collaboration between AZ and IO Informatics ("IO"). AZ provided the project environment, data resources, subject matter expertise ("SME") and business expertise. IO provided semantic software, data modeling and integration services including solutions architecture, knowledge engineering and software engineering.

The project goal was to improve search and retrieve of clinical trials data. SIEVE was to provide a web-based environment suitable for cross-study analysis. The environment was to align across biomarkers, statistics and bioinformatics groups. Over-arching goals included decision-making for biomarker qualification, trial design, concomitant medication analysis and translational medicine.

The team analyzed approximately 42,000 trials records, identified by unique subjectIDs. IO’s Knowledge Explorer software was used by IO’s knowledge engineers in collaboration with AZ’s SMEs to explore the content of these records as linked RDF networks. Robust metadata descriptors were central to the integration. RowID was applied to match entries from a diverse source documents to unique rows in unique study documents. SubjectID and studyID were also important for combining data from separate rows into an integrated resource, for example to combine 2 or more rowIDs specific to a single study subject. Because almost all docs had both subjectID and studyID, concatenation of these two items as an individual identifier allowed connections that bridged multiple documents for data traversal. For data quality assessment, determining the error rate in making connections was possible by evaluating Gender and DOB associated with the concatenated individual identifier. About 6,000 patients could not be associated to both Gender and DOB, and were removed from corpus. Next, as Gender and DOB information were duplicated throughout the corpus, this allowed to test the consistency of data recording, which was reasonably high. Less than 40 individuals had problematic DOB and or gender information where one or more rowIDs did not agree for a subject. In summary, 36,000 records were found to contain valid data that could be usefully linked - each including a unique trial (StudyID), and unique and valid patient (SubjectID), and at least one row of valid laboratory data of interest.

IO created a semantic data model or "application ontology" to meet SIEVE requirements. The resulting data model and instances were harmonized by application of SPARQL-based rules and inference and were aligned with AZ standards. Data was integrated under this ontology, loaded into a semantic database and connected to IO’s "Web Query" software. The result is a web-based User Interface accessible to end users for cross-study searching, reporting, charting and sub-querying. Methods include "Quick Search" options, shared searches and query building containing nesting, inclusion / exclusion, ranges, etc. Advanced Queries are presented as filters for user entry to search subjects ("views" or "facets") including Clinical Assays, Therapy Areas, Adverse Events and Subject Demographics. Reports include exporting, charting, hyperlink mapping and results-list based searches.

Results include reduced time to evaluate data from clinical trials and to facilitate forward looking decisions relevant to portfolios. Alternatives are less efficient. Trial data could previously be evaluated within a study; however there was no method to evaluate trials data across studies without manual intervention. Semantic technologies applied for data description, manipulation and linking provided mission-critical value. This was particularly apparent for integration and harmonization, in light of differences discovered across resources. IO’s Knowledge Explorer applied data visualization and manipulation, application of inference and SPARQL-based rules to RDF creation. This resulted in efficient data modeling, transformation, harmonization and integration and helped assure a successful project.

9.6 Sampling Large RDF Graphs

SampLD is a tool for sampling large RDF graphs. We use the network topology of an RDF graph to detect which triples are 'interesting' enough to be included in a sample. SampLD uses common network analysis tools such as PageRank to detect 'interesting' triples. Because we cannot directly apply pagerank to RDF graphs (as an RDF graph has labelled edges), we first apply a rewrite step where we rewrite RDF to a graph with unlabelled edges (without losing too much information). We determine the quality of the sample by how good a sample is able to return answers to a set of queries (i.e. calculating recall). (This is why we use BioPortal, as we have access to the querylogs via the USEWOD challenge). However, the performance of each sample method (i.e. combination of rewrite method + network analysis algorithm) differs between datasets. The probable reasons for these differences are: (1) the structure of each dataset is different, (2) the queries for each dataset have a different structure. (2), is something which we are currently analyzing by extracting important features from queries. However, (1) is still an open case. What we need are features of an RDF graph. This is where the dataset metrics are useful. They provide an easy way of generating information about the RDF structure itself.

9.7 Query Formulation Using Data Metrics

YASGUI [[YASGUI](https://www.w3.org/TR/hcls-dataset/#YASGUI)] is a query builder for SPARQL with a strong focus on usability. To assist the user in formulating queries, YASGUI provides prefix autocompletion autocompletion using prefix.cc [[Prefix.cc](https://www.w3.org/TR/hcls-dataset/#prefixcc)], endpoint autocompletion using the CKAN-based datahub.io [[Datahub](https://www.w3.org/TR/hcls-dataset/#Datahub)], and property/class autocompletion using the LOV [[LOV](https://www.w3.org/TR/hcls-dataset/#LOV)] API However, none of these autocompletions are based on the users dataset where the query is written for. What users need are autocompletions based on the target dataset, in a fast and scalable manner. However, querying the complete dataset for this information via SPARQL, can be expensive and slow. Additionally, the number of suggestions can be large. The dataset metrics enables fetching such autocompletions in a fast manner, supporting meaningful ranking of the suggestions as well (e.g. by how often a particular class co-occurs with the current predicate).

9.8 Data Providers

**9.8.1 Open PHACTS**

Open PHACTS [[OpenPHACTS](https://www.w3.org/TR/hcls-dataset/#OpenPHACTS)] is an open-source project to build a data integration platform for drug discovery data, called the Open PHACTS Discovery Platform [[Gray et al, 2014](https://www.w3.org/TR/hcls-dataset/#Grayetal2014)]. The platform provides a domain specific API through which the integrated data can be retrieved. A key feature of the Open PHACTS Discovery Platform is that it provides provenance links back to the datasets that it has loaded, allowing the user to discover where each data point has come from. The platform is populated with open data sets including ChEMBL, UniProt, ChemSpider and Wikipathways. The data items within these datasets are related through VoID linksets [[VOID](https://www.w3.org/TR/hcls-dataset/#VOID)]. The linksets are published as part of the delivery of the open platform.

The Open PHACTS Discovery Platform aims to rely on the information in the dataset descriptions in order to enable the automatic loading of data. This requires information about the expected publication frequency of the datasets and details of the distribution files associated with a new release of a dataset, i.e. where the data can be obtained from. Once loaded, the Open PHACTS Discovery Platform computes links between the datasets. These linksets need to be published with adequate provenance information about how they were computed and upon which versions of datasets they are derived. Finally, it is important for us to be able to correctly link back to data providers to give credit and assure our users where our data has come from. To do this correctly, we need to know both human readable and machine readable provenance links to the specific files of the versions of the dataset that have been loaded.

**9.8.2 EBI RDF Platform**

The European Bioinformatics Institute (EBI) is the largest Bioinformatics resource provider in Europe. The recently released RDF platform [[EBI-RDF](https://www.w3.org/TR/hcls-dataset/#EBI-RDF)] presents a coordinated effort to bring together RDF resources from multiple services and databases at the EBI. The EBI invests heavily in the curation and annotation of the source databases to ensure the most up-to-date and accurate information is readily available to the scientific community. Given that the generated RDF is typically the result of a conversions from the source database, it is important that our users understand the relationship between the RDF and the source. To address this we publish detailed provenance for each dataset that includes important information such as version number and release date. This data is available in RDF via content-negotiation from stable dataset URIs and is described using a variety of standard meta-data vocabularies. These dataset descriptions will conform to the recommendations outlined in this document.

**9.8.3 Bio2RDF**

Bio2RDF [[Bio2RDF](https://www.w3.org/TR/hcls-dataset/#Bio2RDF)] is an open-source project to provide Linked Data for the Life Sciences. Bio2RDF defines a set of simple conventions to create RDF(S) compatible Linked Data from a diverse set of heterogeneously formatted sources obtained from multiple data providers. Bio2RDF has developed a registry of over 2000 datasets that acts to provide basic information about bio-datasets and to normalize different identifiers to a common URI scheme. For each dataset, Bio2RDF provides key metadata (title, description, license, rights, publisher, creator, date created) along with a wide variety of statistics to describe the internal contents of each dataset so as to facilitate an understanding of their contents and to use in autocompletion services. Bio2RDF will implement the dataset description guidelines for the description of its datasets, the provenance of the datasets, and dataset statistics.

9.9 Data Catalogs

**9.9.1 BioSharing**

BioSharing [[BioSharing](https://www.w3.org/TR/hcls-dataset/#BioSharing)] is a registry of:

1. community-developed data and metadata reporting standards (including minimum information checklists, ontologies and data formats),
2. policies related to data preservation, managing and sharing; and
3. databases

and the relationships between the three elements (standards, policies and databases) in the life sciences (broadly covering biological, natural and biomedical sciences). Community-driven standardization efforts have worked in different domains to ensure that data and experimental details about how the data was generated are made available in an interoperable way. These are important requirements to enable science reproducibility. The BioSharing catalogue works to serve those seeking information on the existing standards, to identify areas where duplications or gaps in coverage exist and to promote harmonization to stop wasteful reinvention, and developing criteria to be used in evaluating standards for adoption.

BioSharing also provides information about life science data, the tools that can be used to access them, the standards that have been used to generate the data or that can be used to access the data. It follows and extends the information required by the **BioDBCore** reporting guidelines. In particular, BioSharing identifies what reporting guidelines, terminological artifacts and/or exchange formats are considered within a particular dataset. BioDBCore [[BioDBCore](https://www.w3.org/TR/hcls-dataset/#BioDBCore), [Gaudet et al 2010](https://www.w3.org/TR/hcls-dataset/#Gaudetetal2010)] is a checklist, or minimum information standard, including the core attributes for the description of biological databases. It is a community-driven effort overseen by the International Society for Biocuration [[ISB](https://www.w3.org/TR/hcls-dataset/#ISB)], in collaboration with the BioSharing catalogue, which implements the BioDBCore guidelines [[BioDBCore](https://www.w3.org/TR/hcls-dataset/#BioDBCore)]. The BioDBCore checklist main goals involve to compile information on biological databases allowing to survey the current landscape and promote the interoperability of the resources by adoption of of syntactic and semantic standards. The proposed core attributes are listed at the BioDBCore website [[BioDBCore](https://www.w3.org/TR/hcls-dataset/#BioDBCore)].

**9.9.2 Integbio Database Catalog**

Integbio Database Catalog [[Integbio](https://www.w3.org/TR/hcls-dataset/#Integbio)] is a catalog which provides basic information on life science databases according to the uniformed description items such as URLs, database description and biological species to promote circulation of the databases created in Japan. Toward our goal of covering all databases scattered within Japan, we already merged the four ministries' existing database lists into Integbio Database Catalog and also continue a survey to collect information of the databases financially supported by research funds in Japan. The content of Integbio Database Catalog is already available to users as CSV format under the Creative Commons CC0 license. Also, the CSV dataset is translated into RDF and currently exposed through a SPARQL endpoint. We participate in the discussion for the dataset description guidelines to refine the RDF dataset.

**9.9.3 identifiers.org**

Identifiers.org [[Identifiers.org](https://www.w3.org/TR/hcls-dataset/#Identifiers.org)] is a system which provides resolvable and persistent URIs to identify data of interest to the life sciences. It relies upon an underlying Registry which provides detailed information on numerous resources which are crucial to scientific research. It includes details on resolving locations where records for a particular resource may be accessed, defines identifier patterns for each data provider, and reflects accessibility of data by recording the up time for individual resource. The content of the Registry is available as RDF, which is defined using DCAT. The use of such terminologies has been agreed upon by numerous communities, and enables standardised descriptions of such repositories and facilitates data sharing and processing between them.

9.10 Experimental Datasets Use Case

The description of the experimental workflows in a standard way is important to understand the datasets produced and to make them re-usable for further investigations or meta-analysis. The ISA infrastructure [[ISAtools](https://www.w3.org/TR/hcls-dataset/#ISAtools)] provides a tabular format and a set of tools to manipulate it, enabling collecting, curating, managing and re-using datasets in a standards-compliant way.

The ISA syntax provides terminology for describing sample characteristics, technologies and measurements, experimental design, instrument parameters, material and data provenance, inputs and outputs of processes and so on. The syntax relies on the annotation with ontological resources for providing domain-specific semantics. There are also tools for converting the ISA representations into RDF: ISA2RDF [[ISA2RDF](https://www.w3.org/TR/hcls-dataset/#ISA2RDF)] produced by the ToxBank consortium [[ToxBank](https://www.w3.org/TR/hcls-dataset/#ToxBank)] and linkedISA [[Gonzalez-Beltran et al 2014]](https://www.w3.org/TR/hcls-dataset/#linkedISA)produced by the ISA-tools team [[ISAtools](https://www.w3.org/TR/hcls-dataset/#ISAtools)]. In particular, the linkedISA tool extends the representation of experimental workflows with the descriptors compliant with the guidelines presented in this document.

Originally designed for describing high-throughput genomics and post-genomics experiments, used in conjunction with other technologies, the ISA format has also been applied for the description of experiments comparing computational methods [[soapdenovo2](https://www.w3.org/TR/hcls-dataset/#soapdenovo2), [Gonzalez-Beltran et al 2014](https://www.w3.org/TR/hcls-dataset/#GBetal2014)], their inputs and outputs and methods, in the form of references to Galaxy workflows.

The community of users of the ISA metadata tracking framework is the ISA commons [[ISACommons](https://www.w3.org/TR/hcls-dataset/#ISACommons)]. It includes both life science (environmental and biomedical domains) researchers and industrial participants who adopted the ISA format and/or the ISA tools such as ISAconfigurator and ISAcreator for curation and data collection, BII for storage [[Rocca-Serra et al 2010](https://www.w3.org/TR/hcls-dataset/#ISASoftwareSuite)], OntoMaton for annotation [[Maguire et al 2013](https://www.w3.org/TR/hcls-dataset/#OntoMaton)] and Risa for interface with R packages [[Gonzalez-Beltran et al 2014](https://www.w3.org/TR/hcls-dataset/#Risa)].

Within the ISA community, there are examples of public repositories such as EBI Metabolights [[Metabolights](https://www.w3.org/TR/hcls-dataset/#Metabolights)] and data publication platforms such as Nature Publishing Group Scientific Data [[Scientific Data](https://www.w3.org/TR/hcls-dataset/#ScientificData)] and BioMedCentral-BGI GigaScience GigaDB [[GigaScience](https://www.w3.org/TR/hcls-dataset/#GigaScience)].
